# Recognition of prior learning candidates’ experiences in a nurse training programme

**DOI:** 10.4102/hsag.v23i0.1080

**Published:** 2018-06-20

**Authors:** Nomathemba B. Mothokoa, Jeanette Maritz

**Affiliations:** 1 Department of Health Studies, University of South Africa, South Africa

## Abstract

Recognition of prior learning (RPL) in South Africa is critical to the development of an equitable education and training system. Historically, nursing has been known as one of the professions that provides access to the training and education of marginalised groups who have minimal access to formal education. The advent of implementing RPL in nursing has, however, not been without challenges. The purpose of this study was to explore and describe the experiences of RPL nursing candidates related to a 4-year comprehensive nursing training programme at a nursing education institution in Gauteng. An exploratory, descriptive and contextual qualitative research design was undertaken. The research sample comprised 13 purposefully selected participants. Face-to-face individual interviews, using open-ended questions, were used to collect data, which were analysed using Tesch’s approach. Recognition of prior learning candidates experienced a number of realities as adult learners. On a positive note, their prior knowledge and experience supported them in their learning endeavours. Participants, however, experienced a number of challenges on personal, interpersonal and socialisation, and educational levels. It is important that opportunities are created to support and assist RPL candidates to complete their nursing training. This support structure, among others, should include the provision of RPL-related information, giving appropriate advice, coaching and mentoring, effective administration services, integrated curriculum design, and a variety of formative and summative assessment practices.

## Introduction

The apartheid system in South Africa had a negative impact on the education and training systems in the country (South African Nursing Council [SANC] 2009:1). Drastic interventions were required to redress this situation. Given the racially exclusive legacy of apartheid, many individuals did not have the undergraduate qualifications required to enter higher education studies. These individuals were able to gain access only through the process of recognition of prior learning (RPL). The South African Qualifications Authority (SAQA) was responsible for the development of the National Qualification Framework (NQF), which was put in place in 1996 with the aim of transforming the educational system in the country (SAQA 2004). Recognition of prior learning is a process of evaluating knowledge and skills acquired through life experience allowing recognition in formal systems (Miguel, Ornelas & Maroco 2015:179). The NQF objectives relevant to RPL include the provision of access to education and training, and facilitation of redress of former unfair discrimination thereof. Recognition of prior learning emphasises and supports the principle of lifelong learning by encouraging individuals to continuously improve their skills. This article outlines RPL nursing candidates’ experience related to a 4-year comprehensive nursing diploma programme. In the context of this study, an RPL candidate refers to enrolled nurse and enrolled auxiliary nurse students who have formally challenged the RPL process and have obtained credits for access to a 4-year comprehensive nursing diploma programme in one of the Gauteng nursing education institutions (NEIs).

### Recognition of prior learning: A contested discourse and practice

Recognition of prior learning has been a contested discourse and practice for more than a decade with numerous opportunities and challenges. Discourses that are more current include authors such as Letseka and Pitsoe (2014) and Cooper, Ralph and Harris (2017) who argue that academic knowledge is often organised around the dominant and prescriptive view of knowledge. This could lead to a mismatch between the RPL candidates’ experiential knowledge and academic knowledge, or, on the other hand, the candidates’ practice skills and expected professional skills. These tensions and contestations are both conceptual and ideological. Earlier studies established that candidates often found it difficult to ‘de-constitute and reconstitute previous unconscious performance into a codified propositional form’ (Trowler 1996:97).

The pedagogic nature of RPL has also been questioned. Cooper et al. (2017:198) viewed experiential knowledge gained in the workplace or through community engagement as different in character, structure and purpose to formal academic knowledge. In addition, Ralphs (2012) review of the literature showed that the model of RPL practice that had been used over time viewed RPL primarily as an assessment practice. Although assessment is obviously an essential feature of all forms of RPL practice, Cooper et al. (2017:205) asserted that RPL may better be seen as a specialised form of pedagogy which provides tools to navigate knowledge boundaries in and across different learning contexts, within a system characterised by differentiation rather than sameness.

Several authors have written on the potential of RPL being either positively or negatively transformative for candidates. According to Miguel et al. (2015:180), advantages primarily occurred at the personal level of the student in terms of learning, increase in confidence, the realisation of the prior knowledge and skills, the value of the life experience and an enthusiasm for further learning. Furthering the discourse on personal dynamics, Houlbrook (2012:559), however, describes student experiences of RPL in the context of work-based learning as generally being anxious and fearful and that candidates often perceive the world of academic study as alien.

## Problem statement

Nursing has historically been known as one of the professions that provides access to training and education to marginalised groups who have minimal access to formal education (SANC 2013:11). Previously, the requirements for the nursing training programme in South Africa were minimal, and individuals with poorer grades were accepted into the profession. In South Africa, the introduction of RPL was met with much criticism and was challenged by many academics who felt it was an unfair practice. They were concerned that it would compromise the quality of education and training. Academics saw the prospect of admitting large numbers of under-qualified adult students with work-related experience as a threat to the institution’s reputation or an erosion of academic standards (Motaung 2009:78).

According to the Gauteng NEI student affairs’ statistics, the number of RPL candidates failing or terminating studies during the 4-year comprehensive nursing diploma programme was between 12% and 16% higher than non-RPL candidates (Mothokoa 2015:3). It was unclear what RPL nursing candidates’ experiences related to a 4-year comprehensive nursing diploma programme at a NEI in Gauteng were. This study aimed to better understand this phenomenon.

The objective of this research was to explore and describe the RPL nursing candidates’ experience related to a 4-year comprehensive nursing diploma programme.

## Theoretical perspective

The study was viewed from the vantage point of the educational theorist Malcolm Knowles’ principles of andragogy. The seminal text is used as reference. In applying this theoretical perspective to the study, RPL candidates are viewed as adults who bring accumulated life experiences with them to their learning encounters (Knowles 1980:44). Adults enter education events with a large quantity of experience that varies from individual to individual. The heterogeneous life experiences of adults hold several implications for teaching (Knowles 1980:50). Adults like to be given an opportunity to use their existing foundation of knowledge gained from life experience and apply it to their new learning experience (Malone 2014:11). Adult learners prefer to voice their own opinions and have a role in directing their learning. It is also important to note that adult learning is unique and that each individual learns at their own pace and in their own way.

## Material and methods

### Research setting

The research setting was a provincial government NEI in Gauteng, South Africa.

### Research design

This study utilised a qualitative design with an exploratory, descriptive and contextual approach (Creswell 2014:190) in which the aim was to gain an in-depth understanding of RPL nursing candidates’ experience.

### Sampling method

Purposive sampling (Richards 2015:37), which is a non-probability sampling method, was used. The authors deliberately considered which candidates would be able to provide the required experiences and should therefore be included in the study. The inclusion criteria were as follows:

- Recognition of prior learning candidates who applied for and were awarded credits for one or more 4-year nursing programme subjects between 2011 and 2013 and who were at their first, second, third or fourth level of training.
- These RPL candidates were either enrolled nurses or enrolled auxiliary nurses who did not meet the admission requirements for access to the 4-year comprehensive nursing diploma programme and followed the RPL access programme.

The exclusion criteria were all other candidates training for the 4-year comprehensive nursing diploma programme who met the standard admission requirements for access into their diploma studies.

### Data collection

Data were collected through face-to-face interviews. The first author conducted the first three interviews with the second author present; thereafter, the second author continued with the interviews. Thirteen nursing RPL candidates took part in the study. Data saturation (Richards 2015:154) was reached after 10 interviews. An additional three interviews were conducted to ensure that no new themes emerged. All personal past knowledge and theoretical knowledge were bracketed so that full attention could be given to the phenomenon that appeared as consciousness (Giorgio 2008:3). This was achieved by attempting to withhold all prior knowledge and past experiences, which would contaminate the studied phenomenon, by keeping a reflective log (Richards 2015:54).

### Data analysis

All data, including interview transcripts and the reflective logs, were analysed independently by the authors using the descriptive analysis technique by Tesch (in Creswell 2014:198). A consensus discussion was held to verify the final themes.

## Measures of trustworthiness

Strategies employed to ensure the quality of data included the following measures of trustworthiness as described in Egon Guba’s seminal text (Guba 1981): credibility (prolonged engagement, triangulation and reflectivity); applicability (rich descriptions); dependability (code recode procedure and audit trails); confirmability (triangulation and reflectivity); and authenticity (fairness, awareness, understanding, action and empowerment) (Onwuegbuzie, Leech & Collins 2008:8).

## Ethical considerations

Participants could choose whether to participate in this study and could also withdraw from the study without penalty. Informed consent was obtained, and participants indicated the place and time most suitable for the interview to be conducted. The identity of all participants was safeguarded. Findings are described in such a way that participants cannot be identified because of the use of identity codes. Each participant was treated fairly.

Ethical clearance for this study was granted by the University of South Africa (HSHDC/251/2013) and the education institution.

## Findings

The study comprised 13 nursing RPL candidates. This included 2 males and 11 females. During the interviews, two participants were in their third level of study, and five participants were in their 4th year and had recently obtained their final year results. Four participants were excluded from the course because of poor academic performance (terminated). One participant received credits for all his first-year subjects and completed the course within three years. All participants were Africans and their age breakdown was as follows: one participant was between the ages of 20 and 29 years, two were between the ages of 30 and 39 years, eight were between the ages of 40 and 49 years and two were between the ages of 50 and 59 years.

Recognition of prior learning candidates experienced a number of realities as adult learners. On a positive note, their prior knowledge and experience supported them in their learning endeavours. Participants, however, experienced a number of challenges on a personal, interpersonal and educational level. What follows is a discussion of the theme and categories as they emerged from the data analysis of interviews conducted with nursing RPL candidates. Table 1 provides a summary of these findings.

**TABLE 1:** Themes, categories and codes.

Theme	Category	Code
The realities faced by the RPL candidates as an adult learner	Positive aspects related to being an adult learner	Prior knowledge and experience
	Personal challenges	Unexpected and un or underprepared
		Advancing age
		Additional responsibilities
		Anxiety regarding their ability to perform academically
	Interpersonal and socialisation challenges	Challenging family relations
		Relationship with younger students and peers
	Educational strain	Course workload
		English proficiency
		The importance of information technology (IT) skills
		Clinical learning experience

RPL, recognition of prior learning.

### Realities faced by the recognition of prior learning candidates as adult learners

The first theme relates to the realities faced by RPL candidates as adult learners. These include positive aspects as well as a number of challenges.

#### Positive aspects related to being an adult learner

As adult learners, participants had prior nursing knowledge and experience, which meant that they did not enter the programme as a ‘blank slate’. A positive result was that they could combine their work experience with the knowledge gained in the course:

‘As an RPL, I did have light and unlike the people coming straight from home who did not have any knowledge about the subjects. I had to match the things that I am doing at work and the things that the college taught.’ (IP 141106-0013)

Snyman (2013:28) defines mature adults or adult learners as those learners who have left education and gained life and work experience prior to entering education again. Knowles (1980:50) argues that because adults have a richer foundation of experience, they are able to relate new learning encounters to their experience base, which in turn may become the foundation of and a rich resource for learning.

#### Personal challenges

Participants, however, experienced a number of challenges. These challenges were of a personal, interpersonal, socialisation and educational nature. For many RPL candidates, the challenges were unexpected, and they felt un or underprepared. Participants held the perception that their prior experience might be sufficient to ease them through the processes and programme, but were caught off-guard when the reality of the course proved otherwise:

‘I thought it was going to be easy because I’ve been a nurse for twenty eight years, so I thought I was just going to knock it out simply. When I came here, I thought that we were going to be taught those things that I was doing at the hospitals. Coming here … I found things are difficult now and it is as if I am in another world … not in nursing anymore.’ (IP 141105-0011)

‘I didn’t know what RPL meant. We only came here and they told us you…if you want to challenge the exam you can write… these are the subjects, that was all, but they didn’t explain to us what it actually means to challenge the exams. So we didn’t know.’ (IP 141106-0012)

Issues relating to underprepared students or students being unaware of the requirements of programmes are not unique to nursing or the South African context. This sentiment is echoed in research findings worldwide (Glessner 2015:33; Wilson & Lowry 2016:268). In a study conducted by Cantwell and Scevak (2004:143), RPL candidates perceived the theoretical structure of their courses to be much simpler, because of their acquired work experience. The authors, however, raised a concern regarding the students’ perception and underestimation of the course content and the relationship between this belief and less functional learning dispositions and poorer academic outcomes. In this study, participants similarly did not fully understand that an adjustment to higher levels of conceptualisation and critical thinking was required compared to their previous level of study or experiences. This meant that they had to put additional effort into acquiring a specified learning standard.

Several participants experienced their advancing age as adversely affecting their learning processes. They felt that should they have been given an opportunity to study at a younger age, they would have coped better. Their experience was that learning and understanding took greater effort and was more time intensive:

‘Another problem is that they allow you to come to school when you are older. They shouldn’t allow people to wait for a long time before coming to school. It’s difficult to cope ….’ (IP 141105-0012)

‘[*Younger students*] they are better because they are fresh … Fresh from school, now we are old.’ (IP 141106-0015)

‘So, you’ve got to struggle, some of us must read ten times before you understand because the brain is also ageing.’ (IP 141105-0011).

Phipps, Prieto and Ndinguri (2013:13) acknowledged that ability, age and self-efficacy are all factors that contribute to an individual’s perception of how easy or difficult learning would be for them, thus affecting their learning intentions. Brod, Werkle-Bergner and Shing (2013:10) warn that the availability of knowledge on the one hand and control processes allowing access to this knowledge on the other follow extremely different lifetime courses. One might therefore ‘know’ the subject content, but may have difficulty in accessing the information or may need more time or prompts to access the information.

As an (older) adult, there were also additional duties, such as family and financial responsibilities, that arose with being mature of age. Adult learners had more responsibilities, for example, family, friends, work and the need for personal quality time. This may lead to difficulties for an adult to make time for learning. If their life is already demanding, then the learning outcome may be compromised (Fuller, Kuhne & Frey 2011:33):

‘Studying is difficult because you’ve got family. You have to travel home, before you can study, you have to prepare for your children and a husband.’ (IP 141106-0013)

‘… you have more responsibilities.’ (IP 141106-0014)

Participants consequently experienced significant anxiety regarding their ability to perform academically, which caused physical stress and mental distress:

‘… as an RPL student they have more pressure than somebody from not being and RPL because of there are so many things contributing to that stress.’ (IP 141106-0017)

‘There was pressure, there was anxiety. Ja all those things. I had to go to a Doctor, and then he referred me to a psychologist, I ended up being physically not well; my BP (Blood pressure) was uncontrollable … I am taking medication ….’ (IP 141122-0019)

Nursing education and care is generally perceived by most nursing students as demanding and anxiety provoking (Hollenbach 2016:43). The stressful nature of nursing and nurse training often leads to physical problems and emotional stress or depressive symptoms among nursing students (Chen et al. 2015:590). Chen et al. (2015) reported a strong correlation between anxiety and depressive symptoms in nursing students.

#### Interpersonal encounters

The additional responsibilities and anxieties often resulted in a strain on their interpersonal relationships with their spouses as well as fellow students:

‘He would always complain, look at this glass, look at the house, look at the children, he would point at everything, everything was wrong and in a mess.’ (IP 141106-0015)

Naseer (2016) confirmed that female students suffered higher levels of mental stress, environmental stress, social stress and spiritual stress–related symptoms than their male counterparts. This could partially be ascribed to gender stereotyping where woman are expected to carry the burden of household and other social responsibilities.

In addition to strained family relationships, some RPL candidates had challenging relationships with the younger students. A few reported negative experiences with younger students and felt they were excluded through distancing interactions and social discrimination:

‘It’s difficult to cope with a class full of young ones.’ (IP 141105-0012)

‘Our English … though it is the same but it differ, the pronunciation … sometimes we will find it difficult to present in the classroom, because this young ones they will laughing [*sic*] at you, so we feel very slow … small. That is why some of us end up not continuing with the course, being ashamed to be laughed at.’ (IP 141105-0011)

‘The younger ones … will be grumbling … because they understood already so they want to move on and we want the lecturer to go back, so it’s a bit difficult.’ (IP 141126-0021)

Humans are by nature social beings and as such have both intrinsic and extrinsic desires to belong and seek support. This need is often amplified when people move to new and unfamiliar environments. Woman often report feeling isolated and rejected in their pursuit for better or higher qualifications (Ojo 2009:75). Older students may feel marginalised and unwelcome when other students make references to their accent or discourage them from speaking in class, and when their silence is subsequently misinterpreted to mean a lack of intelligence:

In order to manage the perceived or possible marginalisation, some RPL candidates identified peers whom they believed were accepting, who valued them as students, and with whom they negotiated more meaningful personal interactions and support. Positive interpersonal relationships thus formed the foundation for their learning and development. It would seem that participants who realised this and harnessed this potential had an improved learning experience:

‘The four of us we have done it, the four of us we were on the same RPL group knowing that we can’t cope with the other young ones. So we decided no, because with the four, we see we are old and then these people they just go blah … blah … blah …blah so we go step by step, so we have make [*sic*] it, together with *lebona* [with them], we are studying, the three of us, even at home we phone each other so, what *negokasetshwane* [it would not be be same] we have been studying alone.’ (IP 141105-0011)

Not all adult RPL candidates had positive experiences with their peers. Some preferred to associate with the younger students for both psychological and academic support:

‘As RPLs we don’t give each other support, that’s my experience in this college. It’s better to mix with the young ones.’ (IP 141106-0013)

‘Some of them may not regard themselves as falling in the peer of the students, they may continue to be isolated, and they click together as the RPLs. So if they’ve got a negative perception about a particular course, they will share the negativity.’ (IP 141121-0018)

Not all candidates experience adaptation and socialisation in the same way. Ojo (2009) found that some (female) students preferred the informal support that resulted from casual relationships with fellow students. The author warns that this preference could potentially compromise their ability to flourish in the new environment. A negative consequence in this study refers to these candidates being perceived as forming a ‘clique’. Cliques can provide a sense of social cohesion, but members also face the danger of viewing themselves as superior to anyone outside the group, thus reinforcing the marginalisation of others.

#### Educational strain

Participants experience the course load as an additional strain along with the personal and interpersonal issues. They expressed concern regarding the course load and felt the time allocated for content was insufficient. For some candidates, the additional educational burden was too much and they discontinued their studies:

‘They will tell you that this courses it too heavy, they can’t go on with it. The other one just resigned, she said no I can’t cope with this heavy load of X college studying, no, it’s enough she resigned last year.’ (IP 141105-0015)

‘I would say my expectations … I expected the course to be a lot easier, but to my surprise there was too much workload it was not easy there were many challenges, clinical area when you go to the hospitals even at school it was just too much workload.’ (IP 141126-0021)

Curriculum overload and constant demands for change in the education arena can have substantial negative consequences for both students and educators (Campbell 2014). We are in danger of increasing the demands on the curriculum to such a degree that we either fail to teach the basics and essentials, or place additional demands to catch up the overflow in our personal or private time. Dubovieciene and Gublinskiene (2014:143) caution that adult students should not be overloaded, and nurse educators should strike a balance between pre-existing and new knowledge. Overburdening adult students might result in superficial or rote learning.

The RPL candidates were schooled in very different educational contexts and practices. They were often used to rote learning and experienced the new teaching formats as different and unfair:

‘we have attended the Bantu education.’ (IP 141105-0011)

‘Now I encounter the challenges? I think the matter … after all they teach about … so most of us that are RPLs we left school many years ago, then when we come here the method of teaching … I think they use OBE so especially when they ask the questions, some question … when we study … I’m talking about myself, when I’m studying I’m using the study guide, so I’m expecting the question from the study guide whereas they twist the questions, but they took the questions from the study guide but they twisted them.’ (IP 141105-0010)

Many RPL candidates were schooled in a rote-learning educational environment. The challenge with rote learning is that memorisation becomes an end in itself, instead of a means to an end. The learning thus fails to become a building block of critical thinking. Outcomes based education (OBE) is based on the constructivist theory of learning, which argues that individuals build new knowledge by adapting their past experience and knowledge to solve new problems. Within OBE, new knowledge emerges through an active, social process, informed by the use of relevant real-world examples (Halbleib & Jepson 2014:109).

Most participants verbalised having experienced difficulties in mastering the English language. This had a negative impact on written and oral communication and interpretation of meaning:

‘Yes, because of sometimes you … the English part can be difficult because of you can interpret things wrongly in your own way, even in a test you can interpret a question in a wrong way because of English, not just because you don’t know, maybe you studied it but you can’t relate that question to what you know.’ (IP 141106-0017)

‘My first day here at the college I go … I came from X District Hospital. When we finished I go there to the matron and say …told the matron that I am coming back because there they talk only English. I can’t understand. I can’t even go forward to participate. When they say come and present I even hide.’ (IP 141105-0009)

Although black students were able to gain access to higher education at historically white institutions of higher learning in 1994, many institutions use English for teaching and learning – a second language to the majority of these students. Many adult nursing students are products of Bantu Education, a tool used by the apartheid government to ensure that they leave secondary school with poor English skills. This has created a significant impediment in students’ capacity to handle the demands of higher education in English (Sebolai 2013:215).

Language, thinking and, therefore, learning are intimately tied together. Elder and Paul (2004:157) claim that the typical university student cannot deeply comprehend what he or she reads. This problem is more apparent when students receive teaching in their second language. A limited proficiency in a language hampers active communication, which may result in a passive manner of information giving and rote learning, because it is linguistically easier to manage.

In addition to mastering the language skills for participating in the educational aspect, participants also realised the importance of information technology (IT) skills, but verbalised that they lacked this skill, which resulted in them finding it difficult to handle certain aspects of their learning process. They felt disadvantaged when comparing themselves with the younger students who had mastered the IT skills and benefited from that:

‘Mm, I think they’re coping better and then also their understanding and then also their using of the technology things, they are … they can Google everything fast and then unlike me, even if I do have the technology, so it takes me a long time. I’ll ask them, why … where must I go, what must I do? Sometimes I’m boring them but with them they can do … let me say, like the time we were doing the practical, the diagnosis of the diseases and then they will do that … the work using their phone and then it’s easier for them. Unlike me, I have to carry all the books all the time and then it’s time wasting but to them it’s easy.’ (IP 141106-0013)

‘Yes, because most of them they are so exposed to technology, they go Google so many things they gather information from the internet meanwhile somebody who is an RPL, you find that that person cannot even use a computer.’ (IP 141106-0017)

The use of computerised information systems for nursing care plans, patient result management, discharge planning, duty rosters and a range of other activities has become a central focus in nursing practice as well as nursing education (Boore & Deeny 2012:176). With the rapid expansion of electronic learning environments, the need to bridge the gap between the generations of learners is critical (Maxon 2015:11).

An additional educational challenge involves the clinical learning environment. Participants expressed a diverse range of sentiments regarding their learning experiences at various clinical facilities. A participant described how the supportive relationship she had with clinical staff enhanced her confidence and improved her self-directed learning:

‘I have never had any problem with the practical facilities; they were all nice and willing to can teach me because … even the hospitals, I didn’t even have a problem.’ (IP 141105-0015).

Some participants experienced clinical facilities as unsupportive, discouraging and threatening environments, which lead to a lack of confidence and failure to achieve learning objectives:

‘No, another thing at hospitals, the people which we met with them, they have a lot of attitude If they saw the student, they’re relaxed they don’t want to work, and if we talk they say no I’m going to call the college, it makes us to lose hope. They don’t teach us, they don’t assist us.’ (IP 141105-0010)

‘Sisters at clinical facilities are busy with their own things and don’t have time for students. They just expect all the work to be done without supervising students. If you tell them to assist, they scare you and say when your tutors come we will tell them that you don’t want to work.’ (NP 14112822)

Challenges as well as opportunities for growth during clinical placements and clinical experiences are often recorded in studies exploring nurse’s attrition and clinical experiences. Positive experiences include the opportunity to grow both personally and professionally (Moagi, Janse van Rensburg & Maritz 2013:361). The importance of clinical facilitators’ attitudes and competence is, however, paramount and can either ‘make or break’ the students’ experience (Roos 2014:361).

## Conclusions, limitations and recommendations for future research

This study confirms that RPL nursing candidates as adult students are faced with multiple role demands, such as having to balance their educational, work, family, partners’ and children’s needs. In order for them to succeed with their studies, they often make great sacrifices. They also face other challenges in terms of academic and institutional barriers, which make it more difficult for them to cope with these demands. It is therefore of enormous importance that opportunities are created in supporting and assisting these candidates to complete their nursing training. This support structure, among others, should include the provision of RPL-related information, giving appropriate advice, coaching and mentoring, effective administration services, integrated curriculum design, and a variety of formative and summative assessment practices. This study was limited in that it mainly focused on only one government nursing student affairs’ statistics education institution in Gauteng, which is a single setting. This may decrease the generalisability of the findings. Recognition of prior learning candidates in other nursing programmes can also be included in future studies to compare findings from different nursing programmes.

This research did not receive any specific grant from funding agencies in the public, commercial or not-for-profit sectors.
